# Testis Sparing Surgery in Pediatric Testicular Tumors

**DOI:** 10.3390/cancers12102867

**Published:** 2020-10-06

**Authors:** Cezanne D. Kooij, Caroline C.C. Hulsker, Mariëtte E.G. Kranendonk, József Zsiros, Annemieke S. Littooij, Leendert H.J. Looijenga, Aart J. Klijn, Annelies M.C. Mavinkurve-Groothuis

**Affiliations:** 1 Princess Máxima Center for Pediatric Oncology, 3584 Utrecht, The Netherlands; C.D.Kooij-2@prinsesmaximacentrum.nl (C.D.K.); C.C.C.Hulsker@prinsesmaximacentrum.nl (C.C.C.H.); M.E.G.Kranendonk-2@umcutrecht.nl (M.E.G.K.); J.Zsiros@prinsesmaximacentrum.nl (J.Z.); A.S.Littooij-2@umcutrecht.nl (A.S.L.); L.Looijenga@prinsesmaximacentrum.nl (L.H.J.L.); 2 Department of Radiology and Nuclear Medicine, University Medical Center Utrecht, 3584 Utrecht, The Netherlands; 3 Department of Pediatric Urology, University Medical Center Utrecht, 3584 Utrecht, The Netherlands; A.J.Klijn@umcutrecht.nl

**Keywords:** Testis sparing surgery, testicular tumors, pediatric, recurrence, testicular atrophy, algorithm, germ cell tumor

## Abstract

**Simple Summary:**

Testis sparing surgery (TSS) is a safe treatment option in selected cases of testicular tumors in adults, focusing on technical feasibility, oncologic safety, preserving of testicular function, and long-term outcome. This surgical technique is also increasingly being considered in children, as benign tumors are more common in this population. With this systematic review, we aim to evaluate outcome of TSS and to investigate under which circumstances TSS can be considered safe in boys with testicular tumors. Based on the current practice described in this systematic review, combined with the outcome of TSS, we would like to suggest an algorithm to guide clinicians in determining the appropriate surgical treatment in prepubertal patients less than 12 years of age with a testicular tumor. TSS may lead to improved testicular function and quality of life in boys with testicular tumors.

**Abstract:**

Objective: The purpose of this review is to evaluate the outcomes of testis sparing surgery (TSS) and to investigate under which circumstances TSS can be considered a safe treatment option in pediatric patients with testicular tumors. Methods: A database search was performed in Cochrane, Pubmed, and Embase for studies that focused on TSS as treatment for testicular tumors in the pediatric population, excluding reviews and single case reports. Results: Twenty studies, describing the surgical treatment of 777 patients with testicular tumors, were included in the analysis. The majority of pediatric patients with benign germ cell tumors (GCTs) (mean age: 3.7 years) and sex cord-stromal tumors (SCSTs) (mean age: 6.6 years) were treated with TSS, 61.9% and 61.2%, respectively. No cases of testicular atrophy occurred. Four of the benign GCTs, i.e., three teratomas and one epidermoid cyst, recurred. No cases of recurrence were reported in patients with SCSTs. Of the 243 malignant GCTs (mean age: 4.2 years), only one patient had TSS (0.4%). Conclusion: TSS is a safe treatment option for prepubertal patients less than 12 years of age with benign GCTs and low grade SCSTs.

## 1. Introduction

Testicular tumors in the pediatric population are rare. By far, germ cell tumors (GCT) are the main group of pediatric testicular tumors. There are two incidence peaks in children: between 0-4 years and between 15–19 years, often referred to as prepubertal and (post)pubertal age (type I and II), respectively [1]. In the past, prepubertal children and adolescents with testicular tumors were considered together as a pediatric population. However, it is known that GCTs contain fundamentally different categories. Type I testicular GCTs are non-germ cell neoplasia in situ (GCNIS)-associated, resembling benign teratomas and malignant yolk sac tumors (YST). Type II testicular GCTs often contain mixed histology and are malignant and GCNIS-associated [2,3,4]. Sex cord-stromal tumors (SCSTs) encompass a smaller group of testicular tumors and are mostly benign, although malignant transformation is present in about 5% of the cases [5]. Malignant testicular tumors are more common in the (post)pubertal age group, compromising 12% of cancers in adolescents, compared to 4% in prepubertal children [1]. Based on clinical presentation alone, in the absence of elevated serum markers, no distinction between malignancy and benign can be made [6]. In addition to the difference in malignancy rate, another difference occurs in histological type: young children more often present with a type I GCT, while adolescents predominantly have type II GCTs [2,4,7]. Age is therefore described as most the important prognostic risk factor in patients with GCTs according to the largest retrospective evaluation of the MAKEI dataset [4].

The surgical approach of testicular tumors is based on imaging and quantification of tumor markers, to differentiate between benign and malignant. If the tumor is restricted to one testicle, the standard of care has been a radical inguinal orchiectomy (RIO) of the affected testis [8,9]. Trans-scrotal intervention of any type, including fine-needle aspiration, open testicular biopsy, and scrotal orchiectomy, is often referred to as scrotal violation, which causes increased rates of local recurrence compared to RIO [10,11,12]. Testis sparing surgery (TSS) is increasingly being considered as an alternative surgical treatment strategy. TSS is generally performed through an inguinal approach, in which the tumor is enucleated, with a surrounding margin of normal testicular parenchyma [13,14]. In addition, due to rapid analysis of specific markers for GCT and GCNIS in intraoperative frozen section examination (FSE), implementation of TSS is more feasible [15]. TSS may reduce psychological and cosmetic consequences associated with radical orchiectomy [13]. Moreover, it might reduce the risk of impaired fertility [16]. Furthermore, in both pre- and (post)pubertal patients with a solitary testis preoperatively, TSS is recommended to preserve Leydig cell function and thereby testosterone production, and any fertility potential [17]. 

In adults, there is increasing evidence that TSS can be safely performed in cases of benign lesions and carefully selected cases of testicular tumors in terms of technical feasibility, preserving testicular function, as well as respecting oncological surgical margins and preventing long-term recurrence [18,19,20]. Even in bilateral synchronous or metachronous malignant testicular GCTs, TSS could be an option. However in this last case, all patients should undergo adjuvant radiation therapy with 20 Gy to eradicate GCNIS [21]. In the pediatric population, TSS is increasing in prepubertal children, considering the high incidence of benign lesions, as was recently described by Radford et al. [22]. Paratesticular rhabdomyosarcoma and testicular hematolymphoid tumors were not in the scope of this study.

In this systematic review we present the current practice of surgical treatment of testicular tumors and aim to evaluate outcome of TSS and to investigate under which circumstances TSS can be considered as a safe treatment option in pediatric patients with testicular tumors.

## 2. Results

### 2.1. Search and Selection

**Figure 1:**
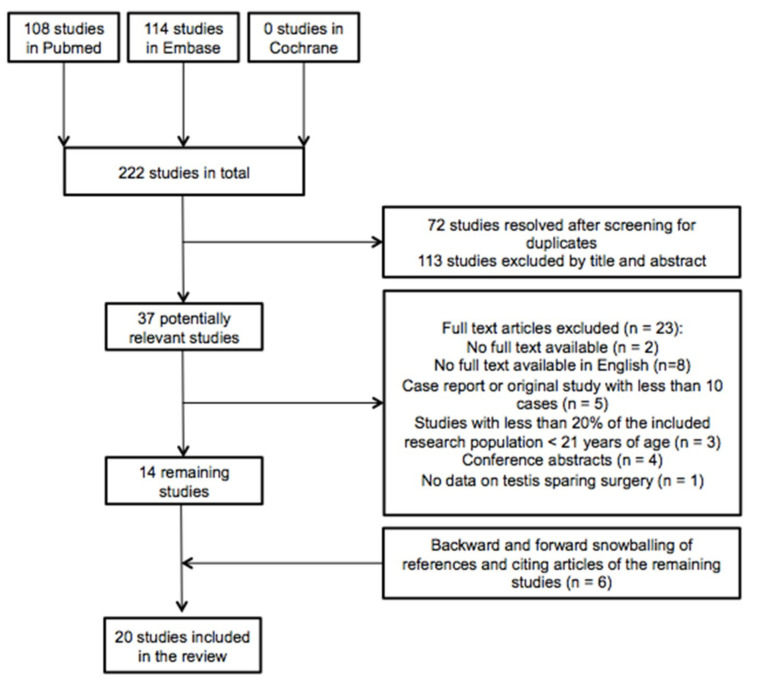
Flowchart of included and excluded articles during the search process.

The database searches of Pubmed, Embase, and Cochrane yielded 222 studies, of which 72 were duplicates. These unique 150 articles were independently screened on title and abstract by the two authors (C.K. and A.M.) based on which 113 studies were excluded. An additional 23 articles were eliminated after full-text review based on the defined exclusion criteria. The 14 remaining articles were analyzed in this review. As a result of manual searching based on snowballing of references and citing studies of the remaining included articles, six more studies were obtained, resulting in a total of twenty articles. Figure 1 gives an overview of the selection and search process based on the PRISMA schema. 

### 2.2. Quality Assessment

An overview of the quality assessment of the 20 included articles, using the STROBE checklist, is provided in Figure S1 (including the STROBE Checklist). 

Additionally, all studies were scored based on their level of evidence, as shown in Table 1. All included articles could be classified as level 4.

### 2.3. Characteristics of the Included Articles

Table 1 shows the characteristics of the 20 included studies. These studies describe a total of 968 patients, of which the surgical treatment of 777 patients with testicular tumors was analyzed. Of the 534 benign tumors, consisting of 444 GCTs (mean age: 3.7 years), 85 SCSTs (mean age 6.6 years), and five miscellaneous tumors (mean age: 1.9 years), 331 (61.0%) were treated with TSS. Of the 243 patients with malignant GCTs, among which 202 YSTs (mean age: 2.0 years) and eight mixed GCTs (MGCTs) (mean age: 5.4 years), one (0.4%) YST was treated with TSS. The number of patients in the study populations of the studies ranged between 11 and 171. Four studies specifically observed a population with benign tumors [27,29,35,36]. One study focused solely on malignant tumors [28]. Thirteen studies specifically examined the prepubertal population [22,24,25,26,27,28,31,33,34,35,37,39,41]. Six articles reported on both pre- and (post)pubertal children, although the majority of their population was prepubertal [21,23,30,32,36,38]. One study has related exclusively to testicular Leydig cell tumors in children and adults [35]. In all studies, TSS is considered for benign pathology only, except for one malignant GCT [28].

### 2.4. Criteria for TSS

The included studies describe the surgical treatment in testicular tumors, based on imaging, serum tumor markers (STMs) and sometimes FSE. As previously mentioned, TSS was considered in benign pathology only. Twelve of the included studies report preoperative or intraoperative findings required for consideration or performance of TSS. Cecchetto et al. describe the presence of normal AFP and hCG levels as the requirement for performance of TSS [32]. Liu et al. performed TSS in patients with negative STMs and typical benign characteristics on US (ultrasonographic) imaging [24]. In the study of Wu et al., normal AFP levels in combination with salvageable testicular parenchyma on US were required for considering TSS [25]. Ye et al. took TSS into consideration in cases with negative STMs and other characteristics of a benign tumor, and in absence of malignancy on FSE, TSS was performed [26]. Of the 17 studies that have described the use of FSE [21,22,23,24,25,26,27,28,29,30,32,33,34,35,36,39,41], nine studies required a benign FSE to perform TSS [22,23,26,28,29,32,33,34,39]. Eight studies incorporated the technical feasibility in the considerations of a testis sparing approach [22,26,28,29,32,33,34,39]. In addition, four studies propose an algorithm for determining the appropriate surgical approach based on US findings, preoperative AFP levels, and FSE [25,28,33,35].

### 2.5. TSS in GCTs

Fifteen of the included studies described cases of benign GCTs, comprising a total of 444 patients of whom 275 (61.9%) were treated with TSS [22,23,24,25,26,27,30,31,32,33,34,35,36,37,38] (Table 2). AFP levels were normal, as expected. Three studies shared their findings on preoperative US, describing teratomas as heterogeneous solid lesions, sometimes in combination with cystic lesions or calcifications, and epidermoid cysts as solitary intratesticular hypoechoic cystic lesions [22,25,32]. Liu et al. describe poor blood flow inside the lesions on Color Doppler flow imaging [24]. 

Testicular atrophy after TSS did not occur in any of the reviewed studies. Local tumor recurrence was found in four patients with a benign GCT; three patients with a teratoma [25,30] and one patient with an epidermoid cyst [27]. One teratoma recurred because of incomplete primary resection and one due to initially wrong diagnosis based on FSE [36]. For one mature teratoma, no reason for recurrence was given [33]. The patient, 15 years of age, who presented with a recurrent epidermoid cyst was initially treated with TSS at age 2 years at another center [27]. 

Thirteen studies (*n* = 243) describe malignant GCT variants, mostly prepubertal YST and to a lesser extent MGCT [22,23,24,25,26,28,30,31,33,34,37,38,41]. In addition, six cases of embryonal carcinoma and one case of choriocarcinoma were reported [30,31]. In contrast to benign GCTs, only one patient with malignant GCT was treated with TSS. This concerned a patient with a stage II YST. No explanation for treatment was indicated and this particular patient was lost to follow-up during the study [28].

### 2.6. TSS in Non-GCTs

Table 3 lists studies that evaluated non-GCTs. The classification of this table is based on the World Health Organization (WHO) 2016 Classification of non-GCTs of the testis and distinguishes SCSTs from miscellaneous tumors [5]. Table 3a shows 12 studies evaluating a total of 85 cases of SCST, including Leydig cell tumors (LCTs), Sertoli cell tumors (SCTs), and juvenile granulosa cell tumors (JGCTs) [23,26,28,29,30,31,33,34,36,37,38,39]. Nine studies reported STMs, which were found to be normal in all cases [26,28,29,30,31,33,34,37,39]. One study exclusively describing Leydig cell tumors reports findings on preoperative US, describing a hypoechoic lesion in the majority of cases [35].

TSS was performed in 52 patients with benign SCSTs, including 44 (78.6%) patients with LCT, six (37.5%) patients with SCT and two (33.3%) patients with JGCT. Only Cecchetto et al. [32] used a staging system (COG) for SCSTs, reporting 10 cases of stage I SCSTs and one case of stage II LCT. None of the studies described adjuvant treatment. No cases of recurrence or testicular atrophy were reported.

Table 3b shows the miscellaneous tumors investigated in five of the included studies [26,27,28,36,37]. STM levels were described as normal [27,37]. Three patients with hemangioma and one patient with an adenomatoid tumor have undergone TSS. Once more, no cases of recurrence or testicular atrophy have been described. Last, six studies also analyzed intra-abdominal testicular gonadoblastoma, in which logically no TSS was performed [24,31,32,33,37,38].

## 3. Discussion

The purpose of this review was to present the current practice of surgical treatment of testicular tumors, evaluate outcome of TSS, and identify under which conditions TSS could be considered a safe treatment option in pediatric patients with a testicular tumor. We identified 20 studies (*n* = 777 patients) evaluating the outcome of TSS in pediatric patients with a testicular tumor. In line with (non-)systematic reviews evaluating TSS in adults, no randomized controlled trials (RCT) have compared TSS and RIO in children [20,40,42]; only retrospective cohort studies and case series were available. The vast majority of the patients analyzed in the included studies were patients less than 12 years of age. 

The twenty included articles in this review represent the full spectrum of intratesticular tumors in children, including benign and malignant pathology. In all studies, treatment with TSS was only been considered in patients with (suspected) benign tumors, based on scrotal imaging, STMs, and the use of FSE. These diagnostic tools are of great importance to distinguish malignant from benign [9,43,44]. Diagnostic developments, such as the application of a direct enzymatic alkaline phosphatase reactive (dAP) test on FSE to identify presence of GCNIS and malignancy, are of great value in the considerations of treatment strategies in testicular tumors [15].

In prepubertal testicular tumors the focus lies on AFP reflecting the YST component, whereas malignant prepubertal tumors that produce hCG are rarely found. Particularly in YSTs, elevated AFP levels are relevant in more than 90% of cases [45]. In this review, all included studies reported AFP levels, showing elevated levels in YSTs and normal levels in SCSTs and in the great majority of benign GCTs. In infancy however, care must be taken not to mistake raised AFP levels for malignancy, as levels may be high as a result of fetal physiological AFP production [2]. Elevated AFP levels can be observed in the first 12 months of life, and therefore age-specific AFP levels should be used to distinguish normal from pathological [46]. Three of the included studies reported this physiological phenomenon in newborn and infants [22,25,34]. 

US is the first-line imaging tool in the investigation of testicular tumors, because of its low cost, wide availability, and high sensitivity for detecting lesions [45,46]. When US shows an inhomogeneous, not-well described lesion with increased internal blood flow malignancy is suspected in contrast to benign lesions that show predominant cystic components with well-defined borders [47]. When US findings appear inconclusive, magnetic resonance imaging (MRI) can contribute to tissue characterization and localization of testicular tumors. Due to its superior soft-tissue contrast and multiplanar capabilities, MRI can serve as an effective diagnostic tool to help distinguish between benign and malignant lesions [48].

Surgical management based on intra-operative FSE has been described as a tool to reliably and critically evaluate testicular masses [49,50]. The accuracy of FSE forms a key component of TSS due to the rapidly and reliably establishment of the diagnosis of malignant GCT [51]. Most studies performed intraoperative FSE and described the importance of application of FSE in their discussion, focusing on presence of malignancy. Use of a dAP test on FSE, which is described as a reproducible and easy tool to diagnose malignancy and GCNIS [15], was not applied or described in the included articles. Surgical management based on technical feasibility, partly determined by the size of the tumor and its location in the testis, is another factor that determines the use of TSS. Technical feasibility should be assessed pre- and intraoperatively in order to perform TSS, determining the tumor size in comparison to the salvageable testicular parenchyma, infiltration in epididymis and rete testis and blood supply to the remaining parenchyma [52].

In both adult and pediatric patients, RIO is considered the recommended surgical approach. However, TSS can be considered in selected benign cases [14]. Radical orchiectomy could cause psychological and cosmetic disadvantages and could risk overtreatment [43]. Additionally, Nord et al. reported reduced Leydig cell function, although this appeared to be compensated by increased pituitary LH production [53]. As the majority of prepubertal tumors appear to be benign, the pursuit of TSS and thereby analysis of the outcomes is even more important. 

This systematic review shows that in the current practice, benign GCTs are treated with TSS in the majority of cases (61.9%) with good outcome; no reported cases of testicular atrophy and four cases of (local) recurrence. Based on the included studies in this review, TSS had not been practiced in the initial surgical treatment of malignant GCTs in the pediatric population. RIO is still the standard of care. Based on this review, no conclusion can be made about the safety of TSS in malignant disease. Among patients with SCST in this review, 74.6% of LCTs and 37.5% of SCTs have been treated with TSS in which no recurrence has been reported. LCTs in children are suggested to associate with a good prognosis and tend to behave less aggressive in children than in adults, which makes TSS a reasonable surgical approach [54]. Even though less is reported on SCTs in the pediatric population, SCTs also tend to behave benign with the exception of SCTs in older patients [2,55]. 

**Figure 2:**
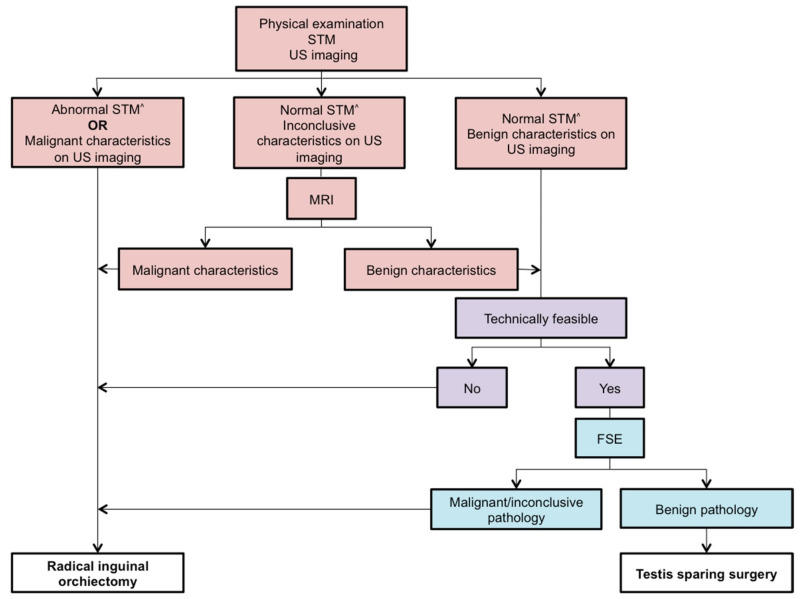
Our proposed algorithm for the surgical approach in prepubertal patients less than 12 years of age with testicular tumors. STM = Serum tumor markers; US = Ultrasonographic; MRI = Magnetic resonance imaging; FSE = Frozen section examination; ^^^ = According to the age specific range; ● = Preoperative; ● = Preoperative and intraoperative; ● = Intraoperative.

Based on the current practice described in this systematic review, combined with the outcome of TSS, we suggest application of an algorithm for determination of the appropriate surgical treatment in prepubertal patients less than 12 years of age with a testicular tumor (Figure 2). We also recommend detection of GCNIS during intraoperative histological examination.

Some limitations are to be noted regarding to this review. First, it has already been stated that no studies used a randomized controlled design. As a result, there was no possibility to perform a quantitative meta-analysis in this review. Furthermore, the differences in sample sizes enabled a dissimilarity of impact on the results per study. In addition, little information has been obtained on (post)pubertal tumors, as most studies focused on the prepubertal population, often with the age limit of 12 years. As a consequence, our results can only be reliably applied to prepubertal patients less than 12 years of age with testicular tumors. The limited inclusion of patients over 12 years of age in the analyzed studies may partly be explained by greater occurrence of malignant type II GCTs. In these patients, TSS may have been poorly or not considered at all. In addition, (post)pubertal patients may also have been reckoned as part of the adult population, while testicular tumors are known for their presence in adolescents and young men. In regard to the malignant tumors in particular, adjuvant therapies have not been included in the report of results in this review. Last, as shown in Figure S1, risk of bias and reasons for loss to follow-up hardly have been described in all studies.

## 4. Materials and Methods

### 4.1. Search Strategy and Study Selection

This systematic review is written according to the checklist of the PRISMA Statement [56]. A systematic search was performed for available published articles up to the 15th of May 2020 in the databases Cochrane, Pubmed, and Embase. These databases were searched for studies that focused on the role of TSS for treatment of testicular tumors in the pediatric population. The main terms that were used in the search and their synonyms are shown in Table S1.

After exclusion of duplicates, all remaining publications were screened on title and abstract by two authors (C.K. and A.M.) independently to identify potential eligible studies. Reviews and single case reports were excluded. After comparison and consensus of the authors on the potentially relevant articles, the remaining publications were screened on the eligibility criteria, based on their full text. 

### 4.2. Eligibility Criteria

Original articles that examined the treatment of testicular tumors with TSS in a pediatric population were considered eligible. Studies with a research population of patients with the age of 21 or less were considered as “pediatric”. Moreover, we decided to only include original studies that included 10 patients or more. In case of original studies with a mixed adult and pediatric population, we decided to only include the study when more than 20% of the study population fell within the pediatric group. The included articles had to be available in full text in English. Both authors have assessed the full texts and disagreements have been resolved by consensus. Manual searching of references was performed based on the Guidelines for Snowballing in Systematic Literature [57]. Only intratesticular tumors were included in this review, excluding paratesticular and testicular hematolymphoid tumors.

### 4.3. Quality Assessment

The checklist of “The Strengthening the Reporting of Observational Studies in Epidemiology” (STROBE) Statement was used to assess the individual quality of reporting of the included studies [58]. The STROBE checklist provides guidelines for the reporting of observational studies. Moreover, the included articles were assessed on level of evidence using the Oxford Center for Evidence-Based Medicine Levels of Evidence Classification rubric by the same two authors [59]. 

### 4.4. Data Collection and Data Items

The following data of the included studies were obtained: author and title, year of publication, population size, number of patients undergoing TSS, median age, serum tumor marker (STM) status (including alpha-fetoprotein (AFP) and human chorionic gonadotropin (hCG), histopathological classification of the testicular tumor, median follow-up time, and primary and secondary long-term outcome (recurrence and testicular atrophy). Outcome of TSS was defined as local recurrence and/or testicular atrophy.

## 5. Conclusions

In conclusion, this review shows that TSS appears to be a safe treatment option for prepubertal patients less than 12 years of age with benign GCTs and low-grade SCSTs. 

## Figures and Tables

**Table 1:** Characteristics of the included articles.

Study	Oxford Level	*n*	TSS (*n*)	Median Age in Years	Median Follow-Up in Months	*n* of Benign Histopathology	*n* of Malignant Histopathology
Caldwell 2019 [23]	4	24	9	10.7	7.15 ^#^	22	2
Liu 2018 [24]	4	171	113	1.66	54	120	51
Wu 2018 [25]	4	67	30	1.5	34	44	23
Ye 2017 [26]	4	47	16	3.17	56	47	0
Friend 2016 [27]	4	21	8	4	ND	12	9
Wei 2014 [28]	4	61	1	1.5	48^^^	0	61
Zahran 2014 [29]	4	13	3	8.7	ND	7	6
Wang 2012 [30]	4	63	15	0.92	50	40	23
Bujons 2011 [31]	4	15	11	8	65	12	3
Cecchetto 2010 [32]	4	11	4	1.92	59 ^^^	ND	ND
Hisamatsu 2010 [33]	4	40	8	1.17	68	25	15
Nerli 2010 [34]	4	22	9	4.6 ^^^	47	16^**^	4 **
Suardi 2009 [35]	4	37	28	33	4.6 years	37	0
Taskinen 2008 [36]	4	34	10	3.2	3.5 years ^#^	23	11
Treiyer 2007 [37]	4	15	3	4	48.2 ^^^	13	2
Shukla 2004 [38]	4	77	13	2.88 ^^^	72	37	40
Metcalfe 2003 [39]	4	48	13	1.5	3 years	27 ^**^	17 **
Ciftci 2001 [40]	4	51	5	3.8^^^	89 ^^^	15	36
Valla 2001 [41]	4	83	56	ND	4.8 years ^^^	83	0
Sugita 1999 [42]	4	68	21	3.6^^^	9 years ^^^	34	22

*n* = Population size expressed as number of testicular tumors; ^^^ = Mean; ^#^ = Follow-up in patients treated with TSS; ND = No (complete/clear) data; ** = Remaining patients had other tumors.

**Table 2:** TSS in benign testicular germ cell tumors.

Study	*n*	*n* TSS (%)	Median Age in Years (Range)	Median Follow-Up in Months (Range)	STM	Recurrence	Testicular Atrophy
Teratoma							
Liu 2018 [24]	87						
	MT (70)	70 (100%)	1.7 (0.4–11.6)	52.2 (20–125)	AFP < 1 years abnormal ^^^^ AFP > 1 year normal, hCG normal	None	ND
	IT (17)	10 (58.8%)	0.4 (0–1)	44.0 (20–115)		None	
Wu 2018 [25]	32	19 (59.4%)	1.6 (0.3–14)	23	AFP normal, hCG normal	None	None
Ye 2017 [26]	37						
	MT (37)	8 (21.6%)	ND	ND	AFP normal, hCG normal	None	None
Friend 2016 [27]	4	1 (25%)	1.5 (0.4–4)	ND	ND	None	ND
Wang 2012 [30]	32						
	MT (27)	9 (33.3%)	0.8	60	AFP > 6 months normal	None	None
	IT (5)	1 (20%)	0.3	26	AFP < 6 months abnormal (2)	None	None
Bujons 2011 [31]	2	2 (100%)	0.3	56.5 (48–65)	AFP normal, hCG normal	None	None
Hisamatsu 2010 [33]	18	5 (27.8%)	ND	ND	AFP < 6 months abnormal ^^^^ (2) AFP > 6 months normal, hCG normal	Recurrence (1)	None
Nerli 2010 [34]	13						
	MT (11)	7 (63.6%)	ND	47 ^#^	AFP normal	None	None
	IT (2)	0 (0%)	ND	ND	AFP normal	None	NA
Taskinen 2008 [36]	16						
	MT (15)	4 (25%)	2.6 (0.2–15.2)	3.5 years (0.5–6.7) ^#^	AFP normal, hCG normal	Recurrence (2)	ND
	IT (1)						
Treiyer 2007 [37]	3						
	MT (2)	0 (0%)	6.5 (4–9)	ND	AFP normal, hCG normal	None	NA
	IT (1)		0.17		AFP normal, hCG normal		
Shukla 2004 [38]	11						
	MT (11)	8 (72.7%)	2.9 (0.3–10) *	88 (5–22) *	AFP normal	None	None
Metcalfe 2003 [39]	22						
	MT (19)	7 (31.8%)	2.3^^^	3 years^^^ (11 months–14 years) ^#^	AFP abnormal (2), hCG normal AFP normal, hCG normal	None	None
	IT (3)					None	None
Ciftci 2001 [40]	9	2 (22%)	2.5 ^^^ (± 0.6)	ND	ND	None	ND
Valla 2001 [41]	33	16 (48.5%)	ND	ND	ND	None	None
Sugita 1999 [42]	27	17 (63.0%)	2.3 ^^^ (0.2–14)	10 years ^^^ (4 months–26)	ND	None	None
Epidermoid cyst		186 + 89					
Liu 2018 [24]	33	33 (100%)	7.1 (0.7–11.8)	50 (18–101)	hCG normal	None	ND
Wu 2018 [25]	9	8 (88.8%)	2 (0.3–13.9)	27	AFP normal	None	None
Ye 2017 [26]	3	3 (100%)	ND	ND	AFP abnormal (1), hCG normal	None	None
Friend 2016 [27]	3	3 (100%)	9 (8–15)	ND	ND	Recurrence (1)	ND
Wang 2012 [30]	4	3 (75%)	10	60.5	AFP normal	None	None
Bujons 2011 [31]	4	4 (100%)	11.5 (10–13)	65 (48–100)	AFP normal, hCG normal	None	None
Hisamatsu 2010 [33]	5	3 (60%)	ND	ND	ND	None	None
Nerli 2010 [34]	3	2 (66.6%)	ND	47 ^#^	AFP normal	None	None
Taskinen 2008 [36]	2	2 (100%)	8.0 (1.2–14.7)	3.5 years (0.5–6.7) ^#^	AFP normal, hCG normal	None	ND
Treiyer 2007 [37]	4	2 (50%)	3.5 (0.1–9)	ND	AFP normal, hCG normal	None	ND
Shukla 2004 [38]	5	5 (100%)	2.9 (0.3–10) **	88 (5–22) **	AFP normal	None	None
Metcalfe 2003 [39]	5	5 (100%)	5.5 ^^^	3 years ^^^ (11 months–14 years) ^#^	AFP < 6 months abnormal (1), hCG normal	None	None
Ciftci 2001 [40]	3	3 (100%)	3.6 ^^^ (± 1.2)	ND	ND	None	ND
Valla 2001 [41]	15	13 (86.7%)	ND	ND	ND	None	ND
Total	444	275 (61.9%)	3.7 ^+^			Recurrence (4)	None

STM = Serum tumor markers; MT = Mature teratoma; IT = Immature teratoma; EC = Epidermoid cyst; AFP = Alpha-fetoprotein; hCG = Human chorionic gonadotropin; ND = No data; NA = Not applicable; ^^^ = Mean; * = Results combined with epidermoid cysts; LR = Local recurrence; ** = Results combined with mature teratomas; ^^^^ = Slightly elevated compared to the age-specific normal range; ^#^ = Follow-up in patients treated with TSS; ^+^ = Mean of median ages.

**Table 3:** (**a**) TSS in testicular sex cord-stromal tumors. (**b**) TSS in miscellaneous testicular tumors.

**(a)**							
**Study**	***n***	***n* TSS (%)**	**Median Age in Years (Range)**	**Median Follow-Up in Months (Range)**	**STM**	**Recurrence**	**Testicular Atrophy**
LCT							
Wu 2018 [25]	1	1 (100%)	6.8	26	AFP normal, hCG normal	None	None
Zahran 2014 [29]	2	1 (50%)	11	24	AFP normal, hCG normal	None	None
Wang 2012 [30]	2	2 (100%)	6	67	AFP normal	None	None
Cecchetto 2010 [32]	4	3 * (75%)	9.8 (6.8–11.6)	59 ^^^ (8–94) **	AFP normal, hCG normal	None	ND
Suardi 2009 [35]	37	28 (75.7%)	33 (5–67)	4.6 years (0.6–16.2)	AFP normal, hCG normal	None	ND
Taskinen 2008 [36]	2	2 (100%)	7 (5.1–8.9)	3.5 years (0.5–6.7) ^#^	AFP normal, hCG normal	None	ND
Treiyer 2007 [37]	1	1 (100%)	6	ND	AFP normal, hCG normal	None	ND
Ciftci 2001 [40]	3	0 (0%)	6 ^^^ (± 0.5)	ND	ND	ND	NA
Valla 2001 [41]	4	4 (100%)	ND	ND	ND	None	None
Sugita 1999 [42]	3	2 (66,6%)	4.8 ^^^ (0.7–6)	15 years ^^^ (12–21)	ND	None	ND
SCT							
Zahran 2014 [29]	2	0 (0%)	3.5	ND	AFP normal, hCG normal	None	NA
Cecchetto 2010 [32]	1	0 (0%)	0.3	59 ^^^ (8–94) *	AFP normal, hCG normal	None	NA
Taskinen 2008 [36]	1	0 (0%)	0.4	3.5 years (0.5–6.7) ^#^	AFP normal, hCG normal	None	NA
Treiyer 2007 [37]	1	0 (0%)	14	ND	AFP normal, hCG normal	None	NA
Metcalfe 2003 [39]	2	0 (0%)	3.1 (0.4–5.8)	ND	AFP normal, hCG normal	None	NA
Valla 2001 [41]	7	5 (71.4%)	ND	ND	ND	None	None
Sugita 1999 [42]	2	1 (50%)	1.2 (0.2–2)	11 years (5–13)	ND	None	ND
JGCT							
Bujons 2011 [31]	1	1 (100%)	10	60	AFP normal, hCG normal	None	None
Cecchetto 2010 [32]	4	1 (25%)	0.3 (0.2–1.9)	59^^^ (8–94) *	AFP normal, hCG normal	None	ND
Valla 2001 [41]	1	0 (0%)	ND	ND	ND	None	NA
Sugita 1999 [42]	1	0 (0%)	2.0	12	ND	None	NA
Undifferentiated							
Cecchetto 2010 [32]	2	0 (0%)	7.2 (0.1–14.3)	59 ^^^ (8–94) *	AFP normal, hCG normal	None	NA
Valla 2001 [41]	1	0 (0%)	ND	ND	ND	None	NA
Total	85	52 (61.2%)	6.6 ^+^			None	
				**(b)**			
**Study**	***n***	***n* TSS (%)**	**Median Age in Years (Range)**	**Median Follow-Up in Months (Range)**	**STM**	**Recurrence**	**Testicular Atrophy**
Hemangioma							
Wu 2018 [25]	1	1 (100%)	1.3	14	AFP normal, hCG normal	None	None
Zahran 2014 [29]	1	0 (0%)	1.3	ND	AFP normal, hCG normal	None	NA
Bujons 2011 [31]	1	1 (100%)	3	24	AFP normal, hCG normal	None	None
Valla 2001 [41]	1	1 (100%)	ND	ND	ND	None	None
Adenomatoid tumor							
Ye 2017 [26]	1	1 (100%)	ND	ND	AFP normal, hCG normal	None	None
Total	5	4 (80%)	1.9 ^+^			None	

STM = Serum tumor markers; AFP = Alpha-fetoprotein; hCG = Human chorionic gonadotropin; ND = No data; NA = Not applicable; ^+^ = Mean of median ages; LCT = Leydig cell tumor; JGCT = Juvenile granulosa cell tumor; ^^^ = Mean; DNT = death not related to tumor; * = 1 scrotal, 2 inguinal; ** = Follow-up in all patients with SCST; ^#^ = Follow-up in patients treated with TSS; ^+^ = Mean of median ages.

## Supplementary Materials

The following are available online at https://www.mdpi.com/2072-6694/12/10/2867/s1, Table S1: Full search strategy in Pubmed and Embase, Figure S1: Quality assessment of the included articles based on the STROBE Statement.
