# Supplementary material for: Testis Sparing Surgery in Pediatric Testicular Tumors
Source: Cancers (Basel). 2020 Oct 6;12(10):2867. doi: 10.3390/cancers12102867 (PMC7600997; doi:10.3390/cancers12102867)
Supplement: Supplementary file 1 [file cancers-12-02867-s001.pdf]

cancers
MDPI

Supplementary Materials

Testis Sparing Surgery in Pediatric Testicular Tumors

Cezanne D. Kooij, Caroline C.C. Hulsker, Mariëtte E.G. Kranendonk, József Zsiros, Annemieke S. Littooij, Leendert H.J. Looijenga, Aart J. Klijn and Annelies M.C. Mavinkurve-Groothuis

Table S1. Full search strategy in Pubmed and Embase.

Pubmed full search	("testicular neoplasms"[MeSH Terms] OR testicular neoplasm*[Title/Abstract] OR testis neoplasm*[Title/Abstract] OR testicular cancer*[Title/Abstract] OR testis cancer*[Title/Abstract] OR testicular germ cell tumor*[Title/Abstract] OR testicular germ cell tumour*[Title/Abstract] OR testicular tumor*[Title/Abstract] OR testicular tumour*[Title/Abstract] OR testis tumor*[Title/Abstract] OR testis tumour*[Title/Abstract] OR "tumor of the testis"[Title/Abstract] OR "tumour of the testis"[Title/Abstract])<br>AND<br>("organ sparing treatments"[MeSH Terms] OR "testis sparing"[Title/Abstract] OR "organ sparing"[Title/Abstract])<br>AND<br>("pediatrics"[MeSH Terms] OR "child"[MeSH Terms] OR "infant, newborn"[MeSH Terms] OR "puberty" [MeSH Terms] OR infant*[Title/Abstract] OR newborn*[Title/Abstract] OR neonate*[Title/Abstract] OR child*[Title/Abstract] OR adolescen*[Title/Abstract] OR teenager*[Title/Abstract] OR youth*[Title/Abstract] OR boy*[Title/Abstract] OR pediatric*[Title/Abstract] OR paediatric*[Title/Abstract] OR prepubert*[Title/Abstract] OR pubert*[Title/Abstract])<br>*Last performed: 15th of May 2020*<br>*Total of 108 results*
Embase full search	('testis tumor'/exp OR 'testicular germ cell tumor'/exp OR 'testicular neoplasm*':ab,ti,kw OR 'testis neoplasm*':ab,ti,kw OR 'testicular cancer*':ab,ti,kw OR 'testis cancer*':ab,ti,kw OR 'testicular germ cell tumor*':ab,ti,kw OR 'testicular germ cell tumour*':ab,ti,kw OR 'testicular tumor*':ab,ti,kw OR 'testicular tumour*':ab,ti,kw OR 'testis tumor*':ab,ti,kw OR 'testis tumour*':ab,ti,kw OR 'tumor of the testis':ab,ti,kw)<br>AND<br>('testis sparing surgery'/exp OR 'organ sparing surgery'/exp OR 'testis sparing':ab,ti,kw OR 'organ sparing':ab,ti,kw)<br>AND<br>('pediatrics'/exp OR 'child'/exp OR 'adolescent'/exp OR 'infant'/exp OR 'boy'/exp OR 'puberty'/exp OR 'infant*':ab,ti,kw OR 'newborn*':ab,ti,kw OR 'neonate*':ab,ti,kw OR 'child*':ab,ti,kw OR 'adolescen*':ab,ti,kw OR 'teenager*':ab,ti,kw OR 'youth*':ab,ti,kw OR 'boy*':ab,ti,kw OR 'pediatric*':ab,ti,kw OR 'paediatric*':ab,ti,kw OR 'prepubert*':ab,ti,kw OR 'pubert*':ab,ti,kw)<br>*Last performed: 15th of May 2020*<br>*Total of 114 results (without MEDLINE)*

STROBE-item	Caldwell 2019	Liu 2018	Wu 2018	Ye 2017	Friend 2016	Wei 2014	Zahran 2014	Wang 2012	Bujons 2011	Cecchetto 2010	Hisamatsu 2010	Nerli 2010	Suardi 2009	Taskinen 2008	Treiyer 2007	Shukla 2004	Metcalfe 2003	Ciftci 2001	Valla 2001	Sugita 1999
1a	●	●	●	●	●	●	●	◐	●	●	◐	●	●	●	●	●	●	●	●	●
1b	●	●	●	●	●	●	●	●	●	●	●	●	●	●	●	●	●	●	●	●
2	●	●	●	●	◐	●	◐	●	●	●	●	◐	●	◐	◐	●	◐	●	●	◐
3	●	◐	◐	◐	◐	◐	◐	◐	◐	◐	◐	◐	◐	●	◐	◐	●	◐	◐	◐
4	●	●	●	●	●	●	●	●	●	●	●	●	●	●	●	●	●	●	●	●
5	●	●	●	●	●	●	●	●	◐	●	●	●	●	●	●	●	●	●	◐	●
6a	◐	●	●	●	◐	●	●	●	◐	◐	◐	◐	●	◐	●	●	◐	●	◐	◐
6b	○	○	○	○	○	○	○	○	○	○	○	○	○	○	○	○	○	○	○	○
7	◐	◐	◐	◐	◐	●	●	◐	◐	◐	◐	●	◐	◐	◐	●	●	●	○	◐
8	●	●	●	●	●	●	●	●	◐	●	●	●	●	◐	●	●	◐	●	◐	◐
9	○	○	○	○	○	○	○	○	○	○	◐	○	○	○	○	○	○	○	○	○
10	●	●	●	●	●	●	●	●	●	●	●	●	●	●	●	●	●	●	●	●
11	●	◐	●	◐	○	●	○	●	○	○	◐	◐	○	○	○	○	○	●	○	○
12a	●	●	●	●	○	●	○	○	○	○	●	○	○	○	○	○	○	●	○	○
12b	●	○	○	○	○	●	○	○	○	○	○	○	○	○	○	○	○	○	○	○
12c	●	○	○	○	○	○	○	○	○	○	○	○	○	○	○	○	○	○	○	○
12d	◐	○	○	○	○	○	○	○	○	○	○	○	○	○	○	○	○	○	○	○
12e	○	○	○	○	○	○	○	○	○	○	○	○	○	○	○	○	○	○	○	○
13a	◐	◐	○	◐	●	◐	●	◐	◐	●	◐	●	●	◐	●	●	●	◐	◐	◐
13b	○	○	○	◐	◐	○	●	○	○	○	○	◐	●	◐	○	◐	●	◐	○	○
13c	○	○	○	○	○	○	○	○	○	○	○	○	○	○	○	○	○	○	○	○
14a	●	●	●	◐	●	●	●	●	●	●	●	●	●	●	●	◐	●	●	●	●
14b	●	●	●	●	●	●	●	●	●	●	●	●	●	●	●	●	●	●	●	●
14c	●	●	●	●	○	●	◐	●	◐	●	●	●	●	●	●	●	◐	●	●	●
15	●	●	●	●	●	●	●	●	●	●	●	●	●	●	●	●	●	●	●	●
16a	●	●	●	◐	○	●	○	○	○	○	◐	○	○	○	○	○	○	◐	○	○
16b	●	●	●	●	○	●	○	○	○	○	●	○	○	○	○	○	○	○	○	○
16c	○	○	○	○	○	○	○	○	○	○	○	○	○	○	○	○	○	○	○	○
17	●	●	●	●	○	●	○	○	○	○	○	○	○	○	○	○	○	○	○	○
18	●	●	●	●	●	●	●	●	●	●	●	●	●	●	●	●	●	●	●	●
19	●	●	◐	◐	○	◐	●	○	○	○	○	○	◐	○	○	○	◐	○	◐	○
20	●	●	●	●	●	●	●	◐	○	◐	●	◐	●	●	●	●	●	●	●	●
21	●	●	●	●	●	●	●	◐	◐	●	●	●	●	◐	●	●	●	●	●	●
22	●	○	●	●	●	○	●	○	○	○	○	●	○	○	○	○	○	○	○	○

● = present; ○ = absent; ◐ = unclear/incomplete

Figure s1. Quality assessment of the included articles based on the STROBE Statement.

<u>STROBE Statement – Checklist of items that should be included in report of observational studies</u>

Title and abstract;

1. Title and abstract
    a. Indicate the study's design with a commonly used term in the title or the abstract
    b. Provide in the abstract an informative and balanced summary of what was done and what was found

Introduction

2. Background/rationale: explain the scientific background and rationale for the investigation being reported
3. Objectives: state specific objectives, including any prespecified hypothesis

Methods

4. Study design: present key element of study design early in the paper
5. Setting: describe the setting, locations, and relevant dates, including periods of recruitment, exposure, follow-up, and data-collection
6. Participants:
    a. Give the eligibility criteria and the sources and methods of selection of participants. Describe methods of follow-up
    b. For matched studies, give matching criteria and number of exposed and unexposed
7. Variables: clearly define all outcomes, exposures, predictors, potential confounders, and effect modifiers. Give diagnostic criteria, if applicable
8. Data sources/measurement: for each variable of interest, give sources of data and details of methods of assessment (measurement). Describe comparability of assessment methods if there is more than one group
9. Bias: describe any efforts to address potential sources of bias
10. Study size: explain how the study size was arrived at
11. Quantitative variables: explain how quantitative variables were handled in the analyses. If applicable, describe which grouping were chosen and why
12. Statistical methods;
    a. Describe all statistical methods, including those used to control for confounding
    b. Describe any methods used to examine subgroups and interactions

c. Explain how missing data were addressed
d. If applicable, explain how loss to follow-up was addressed
e. Describe any sensitivity analyses

Results

13. Participants;
    a. Report numbers of individuals at each stage of study – eg numbers potentially eligible, examined for eligibility, confirmed eligible, included in the study, completing follow-up, and analysed
    b. Give reasons for non-participating at each stage
    c. Consider use of a flow diagram
14. Descriptive data;
    a. Give characteristics of study participants (eg demographic, clinical, social) and information on exposures and potential confounders
    b. Indicate number of participants with missing data for each variable of interest
    c. Summarise follow-up time (eg, average and total amount)
15. Outcome data: report numbers of outcome events or summary measures over time
16. Main results;
    a. Give unadjusted estimates and, if applicable, confounder-adjusted estimates and their precision (eg, 95% confidence interval). Make clear which confounders were adjusted for and why they were included
    b. Report category boundaries when continuous variable were categorized
    c. If relevant, consider translating estimates of relative risk into absolute risk for a meaningful time period
17. Other analyses: report other analyses done – eg analyses of subgroups and interactions, and sensitivity analyses

Discussion

18. Key results: summarise key results with reference to study objectives
19. Limitations: discuss limitations of the study, taking into account sources of potential bias or imprecision. Discuss both direction and magnitude of any potential bias

20. Interpretation: give a cautious overall interpretation of results considering objectives, limitations, multiplicity of analyses, results from similar studies, and other relevant evidence
21. Generalizability: discuss the generalizability (external validity) of the study results

Other information

22. Funding: give the source of funding and the role of the funders for the present study and, if applicable, for the original study on which the present article is based

© 2020 by the authors. Licensee MDPI, Basel, Switzerland. This article is an open access article distributed under the terms and conditions of the Creative Commons Attribution (CC BY) license (http://creativecommons.org/licenses/by/4.0/).
